# Preparation and characterization of artemether-loaded niosomes in *Leishmania major*-induced cutaneous leishmaniasis

**DOI:** 10.1038/s41598-024-60883-0

**Published:** 2024-05-02

**Authors:** Uranous Niroumand, Mohammad Hossein Motazedian, Fatemeh Ahmadi, Qasem Asgari, Mohammad Saleh Bahreini, Parisa Ghasemiyeh, Soliman Mohammadi-Samani

**Affiliations:** 1 Department of Pharmaceutical Nanotechnology, School of Pharmacy, Shiraz University of Medical Sciences, Shiraz-Marvdasht Hwy, Karafarin St, Shiraz, 71468 64685 Fars Iran; 2 Student Research Committee, Shiraz University of Medical Sciences, Shiraz, Iran; 3 Department of Medical Parasitology and Mycology, School of Medicine, Shiraz University of Medical Sciences, Shiraz, Iran; 4 Department of Pharmaceutics, School of Pharmacy, Shiraz University of Medical Sciences, Shiraz, Iran; 5 Pharmaceutical Sciences Research Center, Shiraz University of Medical Sciences, Shiraz, Iran

**Keywords:** Artemether, Niosomes, Topical drug delivery, Cutaneous leishmaniasis, *Leishmania major*, Promastigote, Parasitology, Nanoparticles, Drug delivery

## Abstract

Cutaneous leishmaniasis is the most prevalent form of leishmaniasis worldwide. Although various anti-leishmanial regimens have been considered, due to the lack of efficacy or occurrence of adverse reactions, design and development of novel topical delivery systems would be essential. This study aimed to prepare artemether (ART)-loaded niosomes and evaluate their anti-leishmanial effects against *Leishmania major*. ART-loaded niosomes were prepared through the thin-film hydration technique and characterized in terms of particle size, zeta potential, morphology, differential scanning calorimetry, drug loading, and drug release. Furthermore, anti-leishmanial effect of the preparation was assessed in vitro and in vivo. The prepared ART-loaded niosomes were spherical with an average diameter of about 100 and 300 nm with high encapsulation efficiencies of > 99%. The results of in vitro cytotoxicity revealed that ART-loaded niosomes had significantly higher anti-leishmanial activity, lower general toxicity, and higher selectivity index (SI). Half-maximal inhibitory concentration (IC50) values of ART, ART-loaded niosomes, and liposomal amphotericin B were 39.09, 15.12, and 20 µg/mL, respectively. Also, according to the in vivo study results, ART-loaded niosomes with an average size of 300 nm showed the highest anti-leishmanial effects in animal studies. ART-loaded niosomes would be promising topical drug delivery system for the management of cutaneous leishmaniasis.

## Introduction

Leishmaniasis is an infectious disease caused by protozoan parasites from different species of Leishmania. Leishmaniasis has three main clinical forms including cutaneous leishmaniasis (CL), visceral leishmaniasis (VL), and mucocutaneous leishmaniasis, among which the CL is the most common form^1^. Cutaneous leishmaniasis is mainly caused by *Leishmania tropica*, *Leishmania major*, and *Leishmania aethiopica*^2^. *Leishmania major* (*L. major*) is considered as the most common cause of cutaneous leishmaniasis in the Middle East area^3^. Disease severity can be varied from a self-limited skin lesion (cutaneous leishmaniasis; CL) to lesions spread from the initial skin lesion to the mucosa (mucosal leishmaniasis; ML), or lesions spread through the body uncontrollably (disseminated or diffuse cutaneous leishmaniasis; DCL). DCL causes a potentially fatal systemic disease with multi-organ failure including the spleen, liver, and bone marrow (kala-azar or visceral leishmaniasis; VL)^4,5^. According to the World Health Organization (WHO) reports, 700,000 to 1 million cases of leishmaniasis are diagnosed annually. Moreover, about 200,000 new cases of CL are annually reported to WHO, while since a large number of infected patients usually do not refer to the physician, the real incidence rate is more than 600,000 to 1 million cases annually^6^.

The main therapeutic agents in the management of all clinical forms of leishmaniasis are pentavalent antimonial drugs including meglumine antimoniate (Glucantime®) and sodium stibogluconate (Pentostam®)^7^, which are the drugs of choice for leishmaniasis management based on the WHO report^8^. Other commonly used and clinically available drugs for leishmaniasis management are miltefosine, amphotericin B, pentamidine, and paromomycin. The two major drawbacks of recruitment of these therapeutic agents in leishmaniasis management are their potential toxicities^9^ and the risk of drug resistance occurrence^10,11^.

Artemisinin (from *Artemisia annua* plant) and its derivatives, known as anti-malarial agents, have recently been considered as potential anti-leishmanial agents. Amongst all, artemether (ART) is the most commonly used artemisinin drug for leishmaniasis treatment^12,13^. The anti-parasitic effect of ART is attributed to the endoperoxide bridge in its structure^14^. At first, ART is activated by the intraparasitic heme–iron and cleavage of the endoperoxide bridge which can result in the production of reactive oxygen species which in turn can induce mitochondrial dysfunction and apoptotic-like death of leishmania parasites^14,15^. It has been reported that ART is capable of inhibition of both intracellular and extracellular growth of *L. major*^16^. ART would be used as an efficient drug in leishmaniasis management, however, due to its short half-life (about 3 h), low water solubility, and low bioavailability^17^ due to pre-systemic metabolism, it should be administered frequently which can result in enhanced systemic adverse reactions^13^. Therefore, topical administration of ART for CL management is desirable. Since ART is a highly lipophilic drug, with a logP value of 3.07^18^, and has limited water solubility and low skin permeation, therefore, recruitment of suitable lipid-based nanocarriers including solid lipid nanoparticles (SLNs), nanostructured lipid carriers (NLCs), liposomes, and niosomes with high drug loading capacities for topical drug delivery purposes would be promising^19,20^. To date, ART has been loaded in NLCs^20^, polyvinyl alcohol (PVA) nanoparticles^12^, and nanoemulsions^21^ for CL management. Among various drug delivery systems, niosomes are able to target deeper skin layers and can be used in the treatment of CL, therefore, they were selected in the current study for ART delivery. Moreover, niosomes are capable of targeting macrophages in the deeper skin layers^22^. Niosomes are non-ionic surfactant vesicles with high encapsulation efficiency potential for both hydrophilic and lipophilic drugs. Therefore, they are considered as suitable nanocarriers for drug delivery purposes, especially for topical drug delivery^23^. Numerous anti-leishmanial drugs including amphotericin B^24^, pentamidine^25^, tioxolone^26^, miltefosine^27^, dapsone^28^, and ketoconazole^29^ have been incorporated in niosomes for the purpose of CL management. The main advantages of niosomes over traditional liposomes, as topical drug delivery systems, are their higher encapsulation efficiency, higher physicochemical stability, higher solubilization capacity, enhanced skin penetration due to their more flexible structure, and higher cutaneous permeation capability. In addition, niosomes, can act as a depot for extended drug release purposes. Furthermore, they can induce enhanced therapeutic efficacy of the loaded drug through the delivery to the site of action and also reduce drug clearance^23^. These properties, along with enhanced drug deposition within the target area, sustained and controlled drug release, and reduced systemic absorption, result in reduced adverse drug reactions^30,31^. Various non-ionic surfactants including polyoxyethylene lauryl ether (Brij 35), sorbitan monostearate (Span 60), polyoxyethylene stearyl ether (Brij 72), and polyoxyethylene (80) sorbitan monooleate (Tween 80) are commonly used in vesicular nanoparticles preparation, especially niosomes. The results of a recent study revealed that the niosomes containing Brij 35 showed an enhanced dissolution profile of tacrolimus as a lipophilic agent^32^. The results of another study indicated that the types of surfactants used in niosomes preparation can significantly affect the particle size of the niosomes. In this regard, niosomes containing Tween 60 showed significantly higher particle sizes in comparison to those containing Span 60 or Brij 72. Therefore, niosomes containing surfactants with higher HLB values can result in larger particle sizes^33^.

In this study, ART-loaded niosomes were designed and characterized for topical drug delivery to the deeper skin layers, especially infected macrophages for CL management. At first, the prepared niosomes were optimized in terms of particle size and drug loading. After that, the fabricated optimum niosomal formulations were characterized. Moreover, cytotoxicity and general toxicity assessment were performed on leishmania promastigotes and J774 macrophage cell lines, respectively. In addition, the particle size of ART-loaded niosomes was tuned to achieve optimal cellular intake with specific cell targeting potential with high selectivity index (SI) values. Finally, the in-vivo effect of niosomal ART gel on leishmania lesions was assessed using animal models. The main goal of this study was to enhance the anti-leishmanial effects of ART through its encapsulation within the niosomes as suitable drug delivery systems, especially for topical delivery purposes. To the best of our knowledge, to date, there is no published data regarding the design and development of ART-loaded niosomes for *L. major*-induced CL management.

## Results

### Quantitative analysis

As shown in Supplementary Fig. S1, the results of HPLC method validation for ART analysis revealed that this method could specifically detect and quantify ART at a retention time of 6.2 min at λ_max_ of 205 nm. The results of the logistic regression assessment revealed an R square value of 0.9996, indicating sufficient linearity between ART concentration and area under the curve (AUC). In addition, the method validation results showed acceptable sensitivity for ART analysis in pharmaceutical matrices with a limit of quantification (LOQ) and limit of detection (LOD) of 5 µg/mL and 1.66 µg/mL, respectively. Furthermore, this method presented sufficient intra- and inter-day accuracy and precision which is consistent with the FDA guideline^34^. Both intra- and inter-day accuracy values were between 90 to 110%; also, the intra- and inter-day precisions were < 10%.

### Optimization of ART-loaded niosomes

The results of the preliminary study to obtain the optimum ratio of lipid matrix including triolein, capryol PGMC, and cholesterol are summarized in Supplementary Table S1. Based on the results, the lipid matrix of the optimum formulation (F6) consisted of 25% Capryol PGMC, 25% Triolein, and 50% cholesterol. In this regard, this formulation was considered for further assessment. The amount of ART in the optimum formulation was set to 5% of the total mass of the lipid matrix in which drug expulsion did not occur. Based on the independent variables introduced, the Design Expert software suggested 27 runs. The results of response variables including particle size and %EE are summarized in Table 1. As shown, the particle size of the prepared niosomes was between 94 and 689 nm, and their %EE was between 93.58 and 100%. According to the results, the suggested model (two-factorial interactions (2FI) model) was not significant (P-value of 0.27) for particle size; however, the lack of fit was not significant (P-value of 0.09), indicating the fitness of the model. Moreover, based on the optimization results, there was a significant correlation between the concurrent effect of Brij 72 and Span 60 and the particle size of the niosomes. Based on the optimization findings, two different particle sizes of 100 and 300 nm with the desired %EE were targeted through the Design-Expert and considered as the optimum formulations for further assessments of the effect of niosomes particle sizes. The suggested independent variables by Design-Expert for these targeted particle sizes (100 and 300 nm) and the obtained response variables including particle size and drug loading are summarized in Table 2. Based on the results, the targeted niosomes suggested by the software (with a desirability value of 1) showed acceptable predictability for the particle size. Therefore, although the suggested model for size optimization by Design-Expert was not significant, it showed sufficient repeatability for the suggested runs. In addition, the targeted particle sizes confirmed the predictability of the model. Since the selected ranges for independent variables were narrow, the reported proposed model was not significant. This means that these variables do not have a significant effect on the particle size. Furthermore, the optimum percentage of drug loaded in the lipid matrix (5% of the total mass of the lipids) was accompanied by no drug expulsion or rise in the particle size. Therefore, the suggested model was not significant for %EE, as well.

**Table 1:** Independent variables and response factors of 27 runs suggested by Design-Expert software.

Run no	Independent variables				Response variables	
	Brij 35 (%w/v) [range: 0.5–2%]	Brij 72 (%w/v) [range: 0.5–2%]	Span 60 (%w/v) [range: 0.5–2%]	Surfactant/lipid ratio [range: 0.5–2]	Particle size (nm)	Entrapment efficiency (%EE)
1	1.12	1.30	1.33	2	468	100
2	0.5	0.72	2	0.50	305	100
3	1.20	0.50	2	1.21	332	99.41
4	0.5	2	1.25	0.50	155	100
5	1.29	1.99	1.4	1.15	196	99.51
6	0.50	0.5	0.5	0.73	94	98.70
7	2	0.5	1.25	0.50	298	100
8	1.20	0.5	2	1.21	153	99.24
9	1.29	1.99	1.4	1.15	242	99.42
10	2	1.26	1.25	1.28	108	96.66
11	2	0.5	0.50	2	107	96.22
12	1.85	2	1.25	2	366	100
13	0.50	0.5	1.26	2	111	98.76
14	0.72	1.23	0.62	1.38	689	95.26
15	1.37	1.22	1.70	0.50	138	99.87
16	0.50	2	2	2	104	97.30
17	2	2	0.50	0.50	400	98.30
18	1.21	1.20	0.50	0.50	110	97.56
19	2	2	2	0.50	109	98.79
20	1.12	1.30	1.33	2	435	93.38
21	2	1.26	1.25	1.28	163	97.14
22	0.57	1.35	1.76	1.26	104	95.02
23	2	0.5	2	2	343	94.10
24	0.72	1.23	0.62	1.38	330	99.34
25	0.5	2	0.50	2	293	100
26	1.12	1.30	1.33	2	103	100
27	0.5	0.72	2	0.50	314	99.46

**Table 2:** Targeted runs by design-expert software and the amount of ingredients for artemether (ART)-loaded niosomes with particle sizes of 100 and 300 nm.

Targeted particle size (nm)	Brij 35 (mg)	Brij 72 (mg)	Span 60 (mg)	Capryol PGMC (mg)	Triolein (mg)	Cholesterol (mg)	Surfactant/lipid ratio	Buffer (PBS^1^; ml)	Organic solvent (methanol and chloroform; ml)	Artemether (mg)	Particle size (nm)	Loading capacity (%LC^2^)	Entrapment efficiency (%EE^3^)
100	272.40	932.145	998.49	1101.52	1101.52	2203.04	0.50	20	20	220.30	103 ± 2	3.22 ± 0.04	100 ± 0.10
300	977.24	289.2165	851.31	274.56	274.56	549.13	1.93	20	20	54.91	314 ± 1.5	1.67 ± 0.03	99.46 ± 0.07

^1^Phosphate buffered saline.

^2^Loading capacity.

^3^Entrapment efficiency.

### Characterization of ART-loaded niosomes

#### Particle size, size distribution, and zeta potential analysis

**Figure 1:**
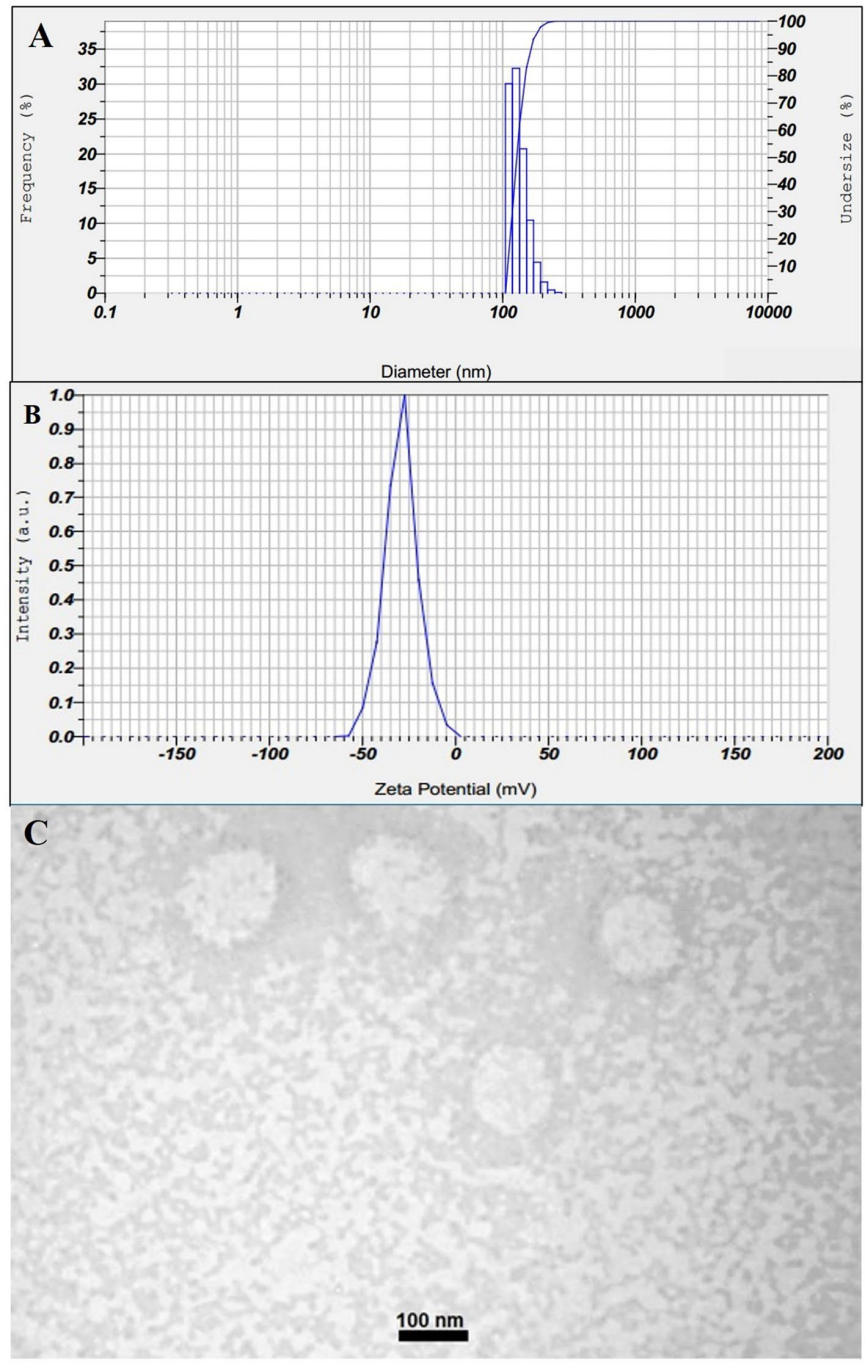
(**A**) Dynamic light scattering (DLS) graph (average particle size of 128.2 ± 2 nm with polydispersity index of 0.266 ± 0.033) (N = 3), (**B**) Zeta potential (− 26.10 ± 3.03 mV) (N = 3), and (**C**) Transmission electron microscopy (TEM) of ART-loaded niosomes with a targeted particle size of 100 nm.

Particle size and size distribution of two optimum ART-loaded niosomes formulations were assessed by static light scattering (SLS) and dynamic light scattering (DLS) techniques. As shown in Supplementary Fig. S2, particle size analyzer (PSA) results revealed that the prepared niosomes had an average particle size of 103 ± 2 nm with a span index of 0.9 and 314 ± 1.5 nm with a span index of 0.266. DLS results were displayed in Fig. 1A, which indicated the polydispersity index (PDI) of 0.266 ± 0.033, showing the homogeneity of the prepared niosomes.

The zeta potential of the prepared ART-loaded niosomes was − 26.10 ± 3.03 mV, as shown in Fig. 1B. This negative zeta potential could induce electrostatic repulsion among the prepared niosomes which could in turn result in higher physical stability profile of the niosomes during storage and avoid aggregation over time.

#### TEM

As shown in Fig. 1C, morphological assessment of the prepared ART-loaded niosomes through TEM revealed that the optimum niosomal formulation was homogenous in size and spherical in shape. In addition, the obtained particle size was compatible with the PSA and DLS results.

#### Drug loading assessment

The results of the drug loading assessment revealed that the %EE of ART within the niosomes with average diameters of 100 and 300 nm were 100 ± 0% and 99.46 ± 0.13%, respectively. Moreover, %LC of ART within the niosomes with average diameters of 100 and 300 nm were 3.22 ± 0.02% and 1.67 ± 0.03%, respectively.

#### Stability assessment

**Figure 2:**
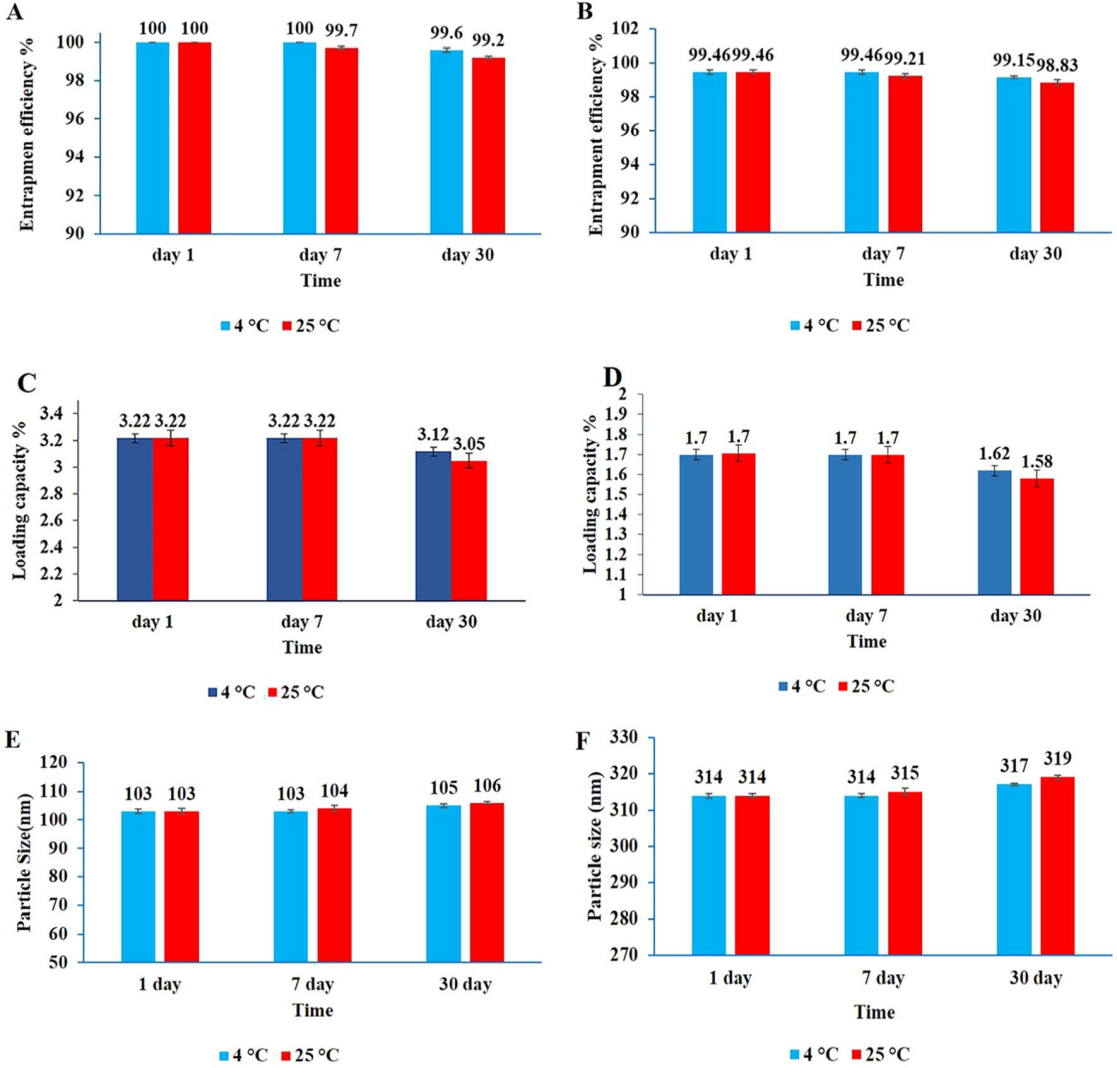
(**A**) Entrapment efficiency (%EE) of artemether (ART)-loaded niosomes with an average diameter of 100 nm, (**B**) %EE of ART-loaded niosomes with an average diameter of 300 nm, (**C**) Loading capacity (%LC) of the ART-loaded niosomes with an average diameter of 100 nm, and (**D**) %LC of ART-loaded niosomes with an average diameter of 300 nm, (**E**) Average particle size of ART-loaded niosomes 100 nm and (**F**) Average particle size of ART-loaded niosomes 300 nm during one month of storage at room temperature (25 °C) and refrigerator (4 °C) (N = 3 for all experiments).

The results of stability assessment in terms of particle size, %EE, and %LC of ART-loaded niosomes with an average diameter of 100 and 300 nm were shown in Fig. 2. Based on the results, the prepared ART-loaded niosomes had acceptable stability, and no significant changes were observed in drug loading (P-values of 0.463 and 0.510 for 100 nm and 300 nm formulations, respectively) and particle size (P-values of 0.585 and 0.418 for 100 nm and 300 nm formulations, respectively) during this period. Furthermore, no drug expulsion occurred during this period of stability assessment.

#### Differential scanning calorimetry (DSC) analysis

**Figure 3:**
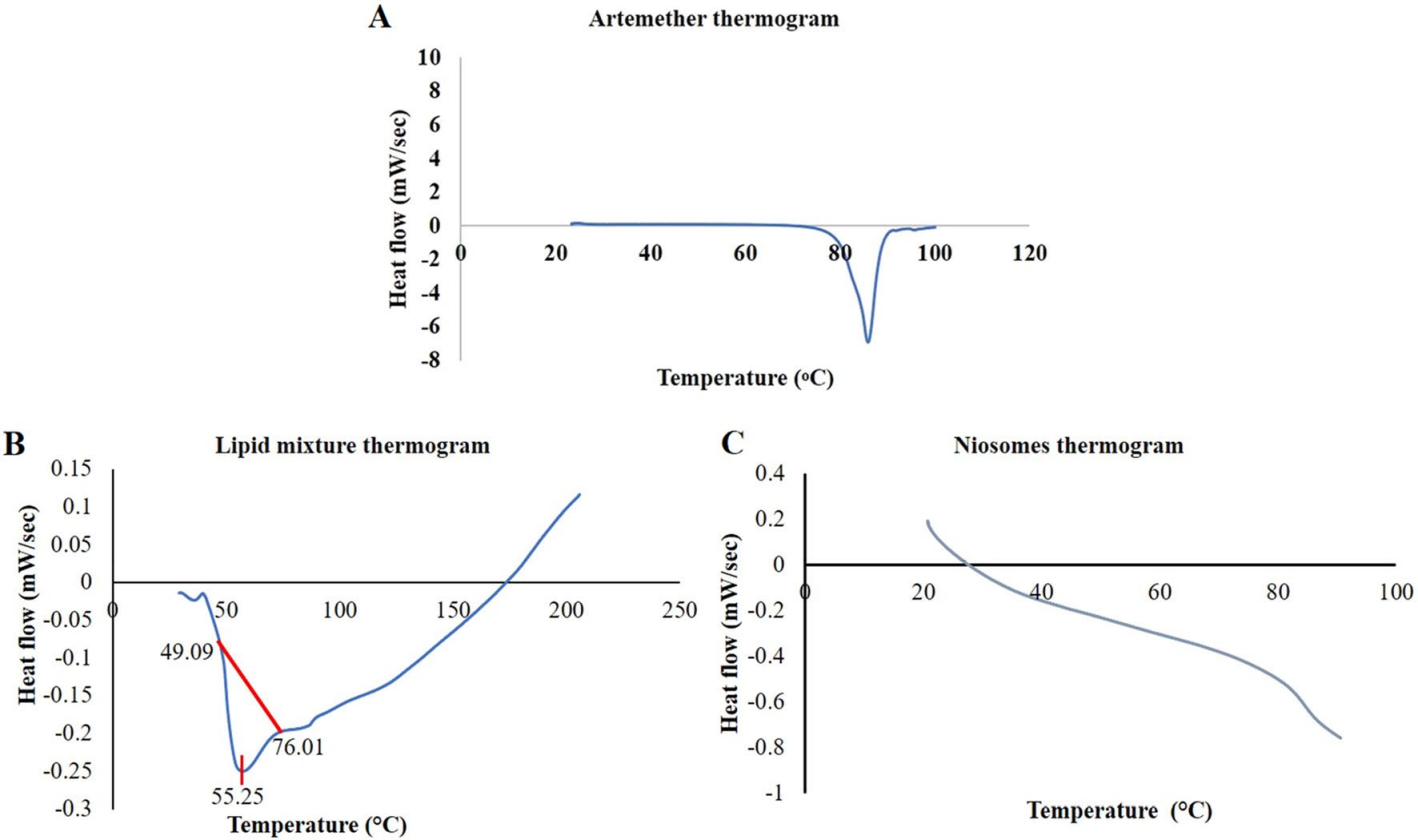
(**A**) Differential scanning calorimetry (DSC) thermogram of artemether; (**B**) DSC thermogram of physical mixture of lipid matrix; and (**C**) DSC thermogram of niosomes with an average diameter of 100 nm.

As shown in Fig. 3, the DSC thermogram of the physical mixture of lipids showed a glass transition temperature (T_g_) of 55.25 °C, while this endothermic peak disappeared in the niosomal formulation, indicating that the prepared niosomes were amorphous and no crystallization was observed. Using surfactants in niosomes formulation profoundly reduces the T_g_. Also, the DSC thermogram of ART was shown in Fig. 3C with an endothermic peak at 85 °C indicating the melting point of ART.

#### Drug release assessment

**Figure 4:**
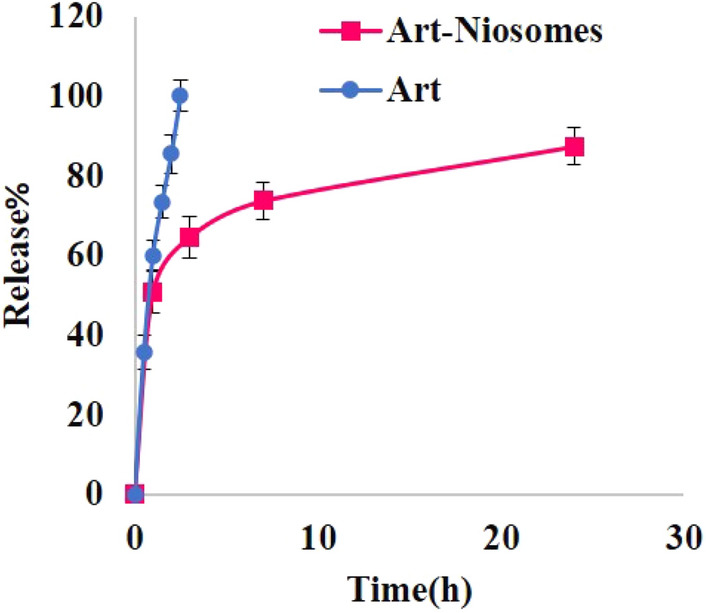
Artemether (Art) release from optimum niosomal formulation (Art-Niosomes) with an average diameter of about 100 nm in comparison to free drug (Art) permeation using centrifugation ultrafiltration technique (N = 3).

The results of cumulative ART release from niosomes and its comparison to the free drug were shown in Fig. 4. Based on the results, about 87.50 ± 4.02% of the total drug was released from the niosomes in 24 h. Drug release from the niosomes followed a biphasic pattern, an initial burst release within the first 2 h followed by a sustained release within 24 h. On the other hand, free drug revealed 100% release within 2.5 h. Therefore, it seems ART encapsulation within the niosomes could result in a more sustained drug release pattern. The results of drug release kinetics from niosomes with an average diameter of 100 nm are summarized in Supplementary Table S2. According to these results, ART release firm the niosomes was best fitted to the First-order model. In addition, the Korsmeyer-Peppas equation was used to distinguish the most probable drug release mechanism from the niosomes. Since the obtained “n” value of the Korsmeyer-Peppas equation (n = 0.169) is < 0.5, therefore, the main mechanism of ART release from the niosomes is Fickian diffusion.

### In vitro anti-leishmanial effects against the promastigotes of *L. major*

**Figure 5:**
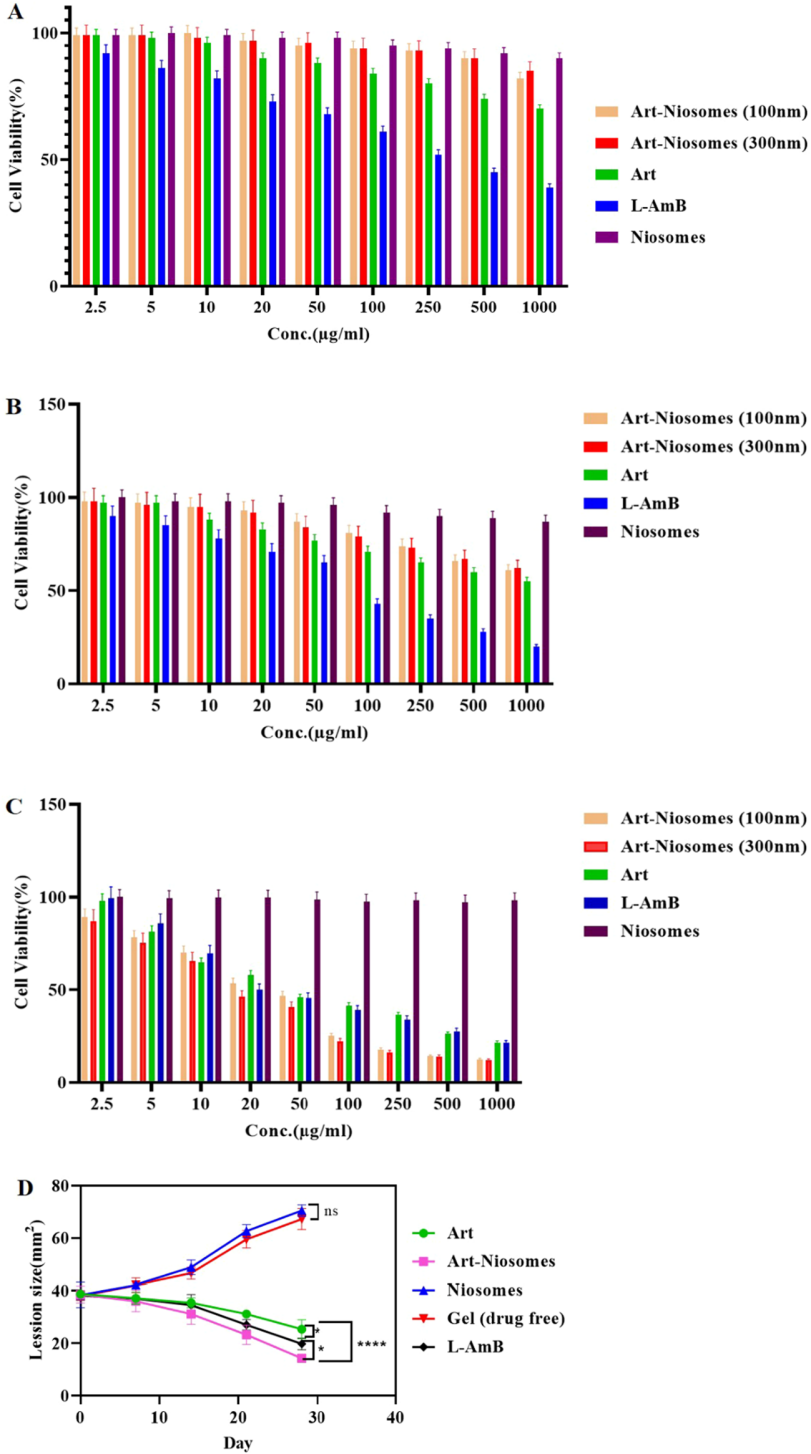
(**A**,**B**) In vitro general toxicity assessment of artemether (Art), ART-loaded niosomes with average diameter of 100 (Art-Niosomes (100 nm)) and 300 nm (Art-Niosomes (300 nm)), drug-free niosomes, and liposomal amphotericin B (L-AMB) at concentration ranges of 2.5–1000 µg/ml against J774 macrophage cell line at (**A**) 24-h and (**B**) 48-h time points. (**C**) In vitro anti-leishmanial effects of ART, ART-loaded niosomes with average diameter of 100 and 300 nm, drug-free niosomes, and liposomal amphotericin B (L-AMB) at concentration ranges of 2.5–1000 µg/ml against promastigotes of *Leishmania major*. (**D**) In vivo anti-leishmanial effects of ART gel (Art), ART-loaded niosomal gel (Art-Niosomes), drug-free niosomal gel (Niosomes), drug-free conventional gel (Gel (drug free)), and topical liposomal amphotericin B (L-AMB) against murine cutaneous leishmaniasis (N = 3 for all experiments).

The anti-leishmanial effects of ART solution, drug-free noisomes, ART-loaded niosomes, and liposomal amphotericin B (L-AMB) at concentration ranges of 2.5 to 1000 µg/ml against the promastigotes of *L. major* (Supplementary Fig. S3) and the percentage of viability were assessed. The half-maximal inhibitory concentration (IC50) values of ART, ART-loaded niosomes with an average particle size of 100 and 300 nm, and L-AMB on promastigotes after 24 h were 39.09, 21.48, 15.12, and 20 µg/mL, respectively, as shown in Fig. 5C and Table 3. Based on the results, the ART-loaded niosomes with an average particle size of 300 nm showed the highest specific cytotoxicity potential against the *L. major* promastigotes in comparison to L-AMB. In this regard, the percentages of cell viability after 24 h were about 25% and 10% for L-AMB and ART-loaded niosomes with an average diameter of 300 nm, respectively. Moreover, the results of 2-way ANOVA statistical analysis revealed that there was a significant difference between the cytotoxic effect of free ART and ART-loaded niosomes in all assessed concentrations against *L. major* promastigotes (P-value < 0.0001). In addition, as shown in Fig. 5, the anti-leishmanial effects of ART-loaded niosomes were significantly higher than those of free ART and drug-free niosomes (P-value < 0.0001 for both). Since there were significant differences in cell viabilities assessed at various concentration ranges from 2.5 to 1000 µg/mL (P-value of < 0.05 for all concentrations), the cytotoxic effect of all evaluated formulations was concentration-dependent and the percentages of cell viability were significantly reduced with concentration enhancement.

**Table 3:** The IC50, CC50, and SI values for free artemether (ART), ART-loaded niosomes with average diameters of 100 nm and 300 nm, and liposomal amphotericin B (L-AMB) as a positive control.

Formulation	Promastigote IC50^3^ (µg/mL)	P-value (in comparison to positive control)	Macrophage cell CC50^4^ (µg/mL)	P-value (in comparison to positive control)	SI^5^
Free ART^1^	39.09 ± 1.66	< 0.0001	1582.00 ± 81.00	< 0.0001	40.47
ART-loaded niosomes 100 nm	21.48 ± 1.20	0.210	4244.00 ± 340.00	< 0.0001	197.57
ART-loaded niosomes 300 nm	15.12 ± 1.13	0.008	4882.00 ± 410.00	< 0.0001	322.88
L-AMB^2^	20.00 ± 1.27	–	244.70 ± 10.00	–	12.23

^1^Artemether.

^2^Liposomal amphotericin B.

^3^Half-maximal inhibitory concentration.

^4^Half-maximal cytotoxicity concentrations.

^5^Selectivity index.

The IC50, half-maximal cytotoxicity concentrations (CC50), and selectivity indices (SI) are presented in Table 3. IC50 showed the minimum concentration of the drug required for half-maximal toxicity against *L. major* promastigotes. CC50 was considered as the minimum concentration of the drug required for half-maximal general toxicity against the intact macrophage cells. SI values showed the selective toxicity potential of the assessed formulation for the promastigotes in comparison to macrophage cells. According to the obtained results, the IC50 values of ART-loaded niosomes with an average diameter of 100 nm and 300 nm were significantly lower than that of ART against the promastigote of *L. major* after 24 h of assessment with P-values of 0.0001 and < 0.0001, respectively. The IC50 value of ART-loaded niosomes with an average diameter of 300 nm was significantly lower than L-AMB as the positive control (P-value of 0.008), while there was no significant difference between the IC50 values of ART-loaded niosomes with an average diameter of 100 nm and L-AMB (P-value of 0.210) against *L. major* promastigotes after 24 h. Moreover, based on the results, the IC50 value of ART-loaded niosomes with particle size of 300 nm was significantly lower than ART-loaded niosomes with particle size of 100 nm (P-value of 0.003) which indicated the size-dependent cellular uptake of the prepared niosomes.

### Cytotoxicity assessment using J774 cell line

The general toxicity of the free drug, ART-loaded niosomes with particle sizes of 100 and 300 nm, drug-free noisomes, and L-AMB as a positive control at concentration ranges of 2.5–1000 µg/ml against macrophage cells (Supplementary Fig. S3) at time points of 24 and 48 h were assessed, and the results were shown in Fig. 5A,B. According to Fig. 5A, the percentage of cell viability at a concentration of 1000 µg/ml for niosomal formulations with particle sizes of 100 and 300 nm after 24 h was about 85%, while L-AMB at the same concentration showed the cell viability of less than 40%. Therefore, the prepared ART-loaded niosomal formulation with the lowest general toxicity in comparison to free ART and L-AMB can be considered as the safest preparation. The CC50 and SI values for ART, ART-loaded niosomes with a particle size of 100 and 300 nm, and L-AMB against macrophages after 24 h are presented in Table 3. The obtained CC50 value of L-AMB was significantly lower than the CC50 values of free ART and ART-loaded niosomes with particle sizes of 100 nm and 300 nm (with P-values of < 0.0001 for all formulations) which confirmed the higher general toxicity of L-AMB. Based on the results, ART-loaded niosomes with an average diameter of 300 nm showed the lowest IC50 of 15.12 µg/mL and the highest CC50 of 4882 µg/mL which resulted in the highest SI value of 322.88. However, the L-AMB had an IC50 value of about 20 µg/mL, CC50 of 244.70 µg/mL, and SI value of 12.23 which was significantly lower than the SI value of ART-loaded niosomes indicating that this new formulation has a more selective anti-leishmanial effect. Therefore, the designed ART-loaded niosomal formulation could specifically induce the highest toxicity against *L. major* promastigotes with the lowest general toxicity potential in comparison to L-AMB and free ART. Although L-AMB showed a suitable IC50 of 20 µg/mL, due to the lower SI value and higher general toxicity, would be inferior to the ART-loaded noisomal formulation.

### In vivo anti-leishmanial effect

According to the results of the in vitro cytotoxicity assay, the ART-loaded niosomes with an average particle size of 300 nm provided the lowest IC50 and the highest SI values; therefore, in the animal study, this niosomal formulation was considered for further assessments. In addition, it seems that these tuned-sized nanoparticles can result in better cellular uptake and internalization by the infected macrophages, which in turn can lead to the higher clinical efficacy of the niosomal formulation.

The *in-vivo* anti-leishmanial effects of conventional ART gel 1%w/w, ART-loaded niosomal gel 1%w/w, drug-free niosomal gel, drug-free conventional gel, and topical nanoliposomal amphotericin B gel 0.4%w/w (SinaAmpholeish®) were assessed, and the results were presented in Fig. 5D. Based on the results, drug-free niosomal gel and drug-free conventional gel were accompanied by an increase in wound size during one month of treatment (Supplementary Fig. S4). While conventional ART gel, ART-loaded niosomal gel, and liposomal amphotericin B resulted in a reduction in the wound size during one month of treatment (Supplementary Fig. S5). Moreover, based on the results, ART-loaded niosomal gel could induce the highest therapeutic effect and result in the smallest wound size after one month of topical treatment. In addition, there was a significant difference between the anti-leishmanial effect of ART-loaded niosomal gel and both conventional ART gel (P-value < 0.0001) and topical liposomal amphotericin B formulation (P-value of 0.01). According to Fig. 5D, after one month of treatment with ART-loaded niosmal gel, the lesion size was reduced to less than 50% of the baseline lesion size which indicates a more efficient anti-leishmanial effect than conventional ART gel (P-value < 0.0001). Therefore, it seems that nisomes due to their flexible structure and the presence of non-ionic surfactants as permeation enhancers, can result in better skin deposition and higher cellular uptake that cause improved anti-leishmanial effect.

## Discussion

The optimum formulation of ART-loaded niosomes with desired particle size was homogenous in size with a spherical shape. The spherical shape of the obtained niosomes was confirmed, using TEM analysis, and the results were compatible with those of simvastatin-loaded niosomes^35^ and also human growth hormone-loaded niosomes^36^. It has been reported that the addition of oils including Triolein and Capryol PGMC can result in a reduction in the particle size of the nanoparticles^37^, therefore, these ingredients were also used to fabricate niosomes with tuned particle sizes. In addition, surfactants can have a pivotal effect on nanoparticles size, as it has been reported, niosomes consisting of Span 60 and Brij 72 (i.e. non-ionic surfactants with lower HLBs) showed significantly smaller particle sizes in comparison to those consisting of Tween 60 with higher HLBs^33^, therefore, it the current study, a mixture of Span 60, Brij 35, and Brij 72 was used to prepare niosomes with the desired particle sizes. The results of the current study revealed that there was a significant correlation between the concurrent effect of Brij 72 and Span 60 and the particle size of the niosomes. Furthermore, it has been reported that niosomes containing Span 60, Span 40, or Brij 72 could induce localized delivery and drug depot within the skin layers for topical drug delivery^38^ which was the main purpose of the current project.

The negative zeta potential of − 26.10 ± 3.03 mV supported the possible higher physical stability of the prepared niosomes due to the induction of electrostatic repulsion and prevention of particle aggregation during the storage period. The negative zeta potential of the prepared niosomes can be attributed to the presence of Span 60 in their formulation which can adsorb hydroxyl ions from the aqueous medium to its surface and induce negative zeta potential^39^. Moreover, it has been reported that cholesterol can significantly affect the zeta potential and electrostatic behavior of the niosomes, in this regard, an increase in cholesterol concentration was associated with a decrease in negative zeta potential value^40^. This negative zeta potential was comparable to the results of previous studies on pilocarpine hydrochloride-loaded niosomes that were fabricated using non-ionic surfactants such as Span 60^41^. In another study, negative zeta potential was obtained for cyclosporine-loaded niosomes for topical drug delivery purposes^42^.

Due to the lipophilic characteristics of ART, with a logP of 3.07, high entrapment efficiency values of 98.78% and 100% for niosomes with an average particle size of 100 and 300 nm, respectively, were predictable. The results of the current study were compatible with those of a previous study reported by Mirzaei-Parsa et al*.* focused on ART-loaded niosomes for breast cancer management which showed an %EE of 82%^43^. The high %EE of ART within the prepared niosomes can also be attributed to the presence of Span 60, as a non-ionic surfactant, in their structure. It has been reported that niosomes comprising Span 60 can induce drug partitioning within the bilayers of Span 60, especially for lipophilic drugs, that in turn can result in enhanced drug loading and increased %EE^44^. Moreover, it has been reported that the relative amounts of Span 60 and cholesterol can significantly affect the drug loading and %EE of lipophilic drugs within the niosomes. In this regard, higher surfactant concentration and lower cholesterol concentration in niosomal formulation were associated with enhanced %EE^45^. The results of our previous study on ART-loaded NLCs showed an %EE of 89.57%^20^; therefore, it seems that the niosomal formulation could enhance %EE of ART in comparison to the NLC formulation. Moreover, the results of this study indicated that the particle size could affect the amount of %EE which was compatible with the findings of a previous study on hydroxycamptothecin-loaded niosomes which reported that an increase in the particle size from 82 to 204 nm could enhance the drug loading capacity of the prepared niosomes^46^.

The stability study revealed no significant changes in %EE, %LC, and particle size of niosomes during one month of storage. These results were comparable to the findings of a previous study reported by Temprom et al*.* described melatonin-loaded niosomes that were composed of Span 60 and cholesterol^47^.

Transmission electron microscopy results revealed that the optimum formulation of ART-loaded niosomes was spherical in shape and homogenous in particle size, being compatible with the data obtained from the PSA and DLS techniques.

According to the results, ART release from niosomes was significantly slower than the free drug. About 100% of free ART was passed through the Amicon filter tubes within 2.5 h, while for ART-loaded niosomes, a biphasic release pattern has been observed, an initial burst release followed by a sustained drug release (a cumulative percentage of 87.5% of the drug was slowly released from the niosomes within 24 h). Therefore, it seems that niosomes are efficiently sustained the drug release pattern in comparison to the free drug, although probably due to the presence of 20% ethanol in the release medium, ART release would be faster than normal condition but due to the sink condition the presence of ethanol in the release medium is essential. The obtained biphasic drug release pattern can be accompanied by longer drug deposition in the skin layers and thereby enhanced localized effect at the wound site, less frequent drug administration, along with minimal adverse drug reactions would be expected. The results of a previous study on topical delivery of adapalene-loaded niosomes revealed an initial burst release (26%) within 1 h followed by a sustained release up to 12 h with a cumulative percentage of 73% drug release^48^. Drug release kinetics revealed that ART release from the niosomes was best fitted with the First-order equation. Moreover, the n < 0.5 in the Korsmeyer-Peppas equation revealed that the Fickian diffusion is the most probable mechanism of ART release from the niosomes. The obtained results were compatible with the results of a previous study on 8-methoxypsoralen-loaded niosomes for topical administration in psoriasis management in which drug release followed a biphasic pattern and the kinetics of drug release was best fitted to the First-order model, also, the according to the obtained “n” value, the drug release mechanism was mainly Fickian diffusion^49^. The same results for drug release kinetics and mechanism of drug release were also reported for topical ethionamide and D-cycloserine-loaded niosomes for tuberculosis management^50^.

According to the results of the MTT assay, ART-loaded niosomes with a particle size of 300 nm showed the highest specific toxicity against *L. major* promastigotes at a concentration of 1000 µg/ml during 24 h of treatment. The SI value for ART-loaded niosomes with a particle size of 300 nm was higher than 100 nm, and SI values for both of these formulations were much higher than that of L-AMB and free ART, indicating that the prepared ART-loaded niosomal formulation can target the *L. major* more specifically and efficiently. Therefore, the cell toxicity of the assessed formulation was concentration-dependent and size-dependent. MTT test results on different therapeutic regimens against *L. major* promastigotes after 24 h indicated a significant difference between ART-loaded niosomes, in both particle sizes and free ART (P-value < 0.05). The IC50 value of the optimum formulation (ART-loaded niosomes with an average diameter of 300 nm on promastigotes after 24 h was calculated as 15.12 µg/mL, however, the results of a previous study on glucantime encapsulated in noisomes against cutaneous leishmaniasis (CL) revealed an IC50 value of 690.8 ± 37.9 μg/mL^24^.

J774 macrophage cell line was used to investigate the general toxicity of different formulations. ART-loaded niosomes showed significantly lower general toxicity in comparison to free ART and L-AMB. Based on the results, ART-loaded niosomes with a particle size of 300 nm showed the lowest level of general toxicity with cell viability of more than 85% after 24 h (Fig. 5A). The SI value for the current study was 322.88 for ART-loaded niosomes with a particle size of 300 nm, while the results of a previous study by Mostafavi et al*.* who prepared amphotericin B in combination with selenium-loaded noisomes revealed the SI value of 288.43^51^. In addition, the results of our previous study on ART-loaded NLCs at lower concentrations showed an SI value of 56.03 after 24 h^20^, while the current study on ART-loaded niosomes resulted in a much higher SI value of 322.88. Therefore, these results can support the superiority of the noisomes over the NLCs for both drug loading and selective and efficient ART delivery to *L. major* promastigotes.

Based on the quantitative assessment of the wound size during the animal study, there was no difference in the wound size between the groups during the first week of treatment (P-value ˃0.05). However, from the second week of treatment, the group that received ART-loaded niosomal gel showed a significant reduction in the wound size compared to the other groups. On the other hand, the mean sizes of the wounds in the negative control groups (drug-free conventional gel and drug-free niosomal gel) were significantly increased (P-value < 0.0001 for both negative control groups) during the treatment courses. In general, the group treated with ART-loaded niosomal gel was significantly accompanied by the highest wound healing capability in comparison to the free drug (P-value < 0.0001) and L-AMB (as positive control) (P-value of 0.01). In the current study, liposomal amphotericin B was considered as the positive control since, according to the WHO guideline, liposomal amphotericin B is one of the approved drugs in leishmaniasis treatment^8^. Moreover, the results of a previous study on the effect of topical liposomal amphotericin B 0.4%w/w against the murine model of cutaneous leishmaniasis revealed a significant reduction in the size of lesions 8 weeks after initiation of treatment in comparison to the negative control group^52^.

In conclusion, a novel topical drug delivery system consisting of ART-loaded niosomes were designed, optimized, and characterized. The optimum niosomal formulations showed the desired particle size of 300 nm with negative zeta potential and reasonable %EE. The in-vitro study of the ART-loaded niosomes by MTT assay showed the superiority of niosomal formulation with an average particle size of 300 nm in comparison to the free drug (ART) and L-AMB. The niosomal formulation showed the highest specific toxicity against *L. major* promastigotes with the lowest general toxicity against the intact macrophage cells. Moreover, in-vitro drug release assessment revealed a sustained release pattern which can result in less frequent drug administration. The *in-vivo* animal study results revealed that the ART-loaded niosomal gel formulation with a particle size of 300 nm was the most effective formulation for decreasing the leishmanial infected wound sizes. Therefore, it seems that the designed ART niosomal gel, as a novel topical drug delivery system, could be promising in the treatment of cutaneous leishmaniasis caused by *L. major.* However, further clinical assessment is needed to show the clinical superiority of this niosomal ART formulation in cutaneous leishmaniasis management.

## Methods

All experiments were performed in accordance with relevant guidelines and regulations.

### Materials

Cholesterol was purchased from Merck, Germany. Brij 35, Brij 72, Span 60, and glyceryl trioleate were obtained from Sigma-Aldrich, St. Louis, Missouri, USA. Capryol PGMC was from Gattefossé, Lyon, France. Dimethyl sulfoxide (DMSO) was purchased from Sentmenat, Spain. 3-(4.5-dimethylthiazol-2-yl)-2.5-diphenyl tetrazolium bromide (MTT), Dulbecco's Modified Eagle Medium (DMEM), and fetal bovine serum (FBS) were obtained from Sigma-Aldrich, USA. Roswell Park Memorial Institute (RPMI)-1640 medium (with L-glutamine and HEPES) was purchased from Shelmax Co., China. Liposomal amphotericin B (AmBisome®) was from Gilead Sciences Co., California, USA. Topical nanoliposomal amphotericin B (SinaAmpholeish) was from Exir Nano Sina Co., Iran. Artemether standard powder was obtained from Santa Cruz, USA. Penicillin and streptomycin were from Gibco, USA. Chloroform, methanol, acetonitrile, and other solvents were HPLC grade and purchased from a local supplier of Merck, Germany. *L. major* standard strain (Mcan/IR/07/Moheb/-gh) was obtained from the Department of Parasitology and Mycology, Shiraz University of Medical Sciences, Shiraz, Iran. Murine macrophage cells (J774 cell line) were obtained from the Department of Immunology, Shiraz University of Medical Sciences, Shiraz, Iran.

### Statistical analysis

In this study, statistical analysis was performed using Design-Expert software (version 10.0.7, Stat-Ease Inc., Minneapolis, USA) and SPSS software (version 26); A P-value of < 0.05 was considered as significant.

### Quantitative determination of Artemether

Artemether analysis was performed through a validated reverse-phase high-performance liquid chromatography (RP-HPLC) method, using Agilent HPLC instrument (Agilent technology 1260 Infinity, USA) equipped with a UV detector. The mobile phase consisted of acetonitrile: water (with a ratio of 75:25%v/v). The flow rate was 1 mL/min, column temperature was fixed at 25 °C, and λ_max_ was set at 205 nm.

### Preparation of ART-loaded niosomes

Niosomes were prepared through a modified thin film hydration technique^14,53^. In this regard, lipid mixture containing triolein, Capryol PMGC, and cholesterol (with a ratio of 25:25:50%w/w); surfactants including Brij 35, Brij 72, and Span 60 (with a ratio of 1.2:1.2:0.5%w/w, and a surfactant/lipid ratio of 0.5:1); and ART (5% w/w of lipid mixture) were dissolved in a mixture of methanol and chloroform (1:1%v/v). Then, the solvent was evaporated at 50 °C and 60 rpm through the vacuum of a rotary evaporator. After that, a dried thin film layer was formed on the inner wall of the flask. The dried thin film was then hydrated using 10 mL of phosphate buffer saline (PBS pH 7.4) at 50 °C and 100 rpm for 30 min to fabricate the niosomal formulation. Finally, the sample was sonicated in 3 cycles of 5 min to achieve a uniform particle size distribution^54^.

### Optimization of ART-loaded niosomes

Formulation optimization was performed by the response surface optimal design using Design-Expert software (version 10.0, Stat-Ease Inc., Minneapolis, USA) according to our previous studies using the same methodology in the optimization process^14,55^. Four independent variables including (1) surfactant to lipid (S/L) ratio, (2) percentage of Brij 35, (3) percentage of Brij 72, and (4) percentage of Span 60 were considered in the optimization process. Response variables were particle size and entrapment efficiency (%EE). According to the defined independent variables, the software suggested 27 runs, as shown in Table 1. Finally, based on the optimization results, ART-loaded niosomes with average diameters of 100 and 300 nm and desired %EE values were targeted by Design-Expert® software (version 10.0.7, Stat-Ease Inc., Minneapolis, USA) for further characterization tests. The rationale for the selection of ART-loaded niosomes with particle sizes of 100 nm and 300 nm through the Design-Expert software was that according to the previous studies, nanoparticles with an average diameter of about 100–300 nm can efficiently enhance the skin permeation of the loaded drug^25,56,57^, which was the main purpose of the current study for CL management.

### Characterization of ART-loaded niosomes

The optimized ART-loaded niosomes were characterized in terms of particle size, size distribution, morphology, physicochemical stability, drug loading, drug release, and differential scanning calorimetry.

#### Particle size, size distribution, and zeta potential analysis

The particle size and size distribution of the freshly prepared niosomes were analyzed through two different methods including: (1) static light scattering (SLS) technique using the particle size analyzer (PSA; SHIMADZU, SALD-2101, Japan) and (2) dynamic light scattering technique (DLS) technique using Microtrac (ZC007, Germany). The span index was calculated according to Eq. (1) to assess the polydispersity and homogeneity of the prepared nanoparticles. In addition, the zeta potential of the prepared ART-loaded niosomes was assessed using Zeta-Chek (Microtract, ZC007, Germany).

1 $$Span index=\frac{D90-D10}{D50}$$

where D_90_, D_50_, and D_10_ are 90%, 50%, and 10% under-sized niosomes diameters, respectively.

#### Drug loading assessment

Drug loading was assessed through the centrifugation ultrafiltration technique using Amicon filter tubes (MWCO 3KDa, Amicon Ultra-4, Millipore Co., MA, USA)^14^. In this regard, 5 ml of the prepared ART-niosomes was poured into the upper chamber of the Amicon® filter tubes. The samples were centrifuged at 4000 rpm for 15 min, and the filtrate was assessed according to validated HPLC method. Furthermore, entrapment efficiency (%EE) and loading capacity (%LC) were estimated using Eqs. (2) and (3), respectively.

2 $$\%EE=\frac{Loaded drug}{Total drug}\times 100$$

3 $$\%LC=\frac{Loaded drug}{Total weight of niosome+Loaded drug}\times 100$$

where, the loaded drug (mg) is the total drug minus the unloaded drug, the total drug is the initial amount of the drug that is used to prepare the niosomes; also, the total weight of niosome (mg) is the initial weight of lipid mixture and surfactants used in the preparation of niosomes. Unloaded drug levels were also calculated through the analysis of the obtained filtrate from the Amicon® filter tubes using the validated HPLC method.

#### Stability assessment

The stability of the optimum ART-loaded niosomes formulations (100 and 300 nm) was assessed in terms of particle size, drug loading (%EE and %LC), and possible drug expulsion^14^. In this regard, the samples were stored both at room temperature (25 °C) and in the refrigerator (4 °C) for up to one month and samples were assessed regularly.

#### Transmission electron microscopy (TEM)

The morphology and particle size of the prepared ART-niosomes were assessed by TEM (Philips, Leo 906E, Germany, voltage of 80 kV). Briefly, the prepared ART-niosomes were fixed on copper grids and stained with uranyl acetate to visualize the niosomes.

#### Differential scanning calorimetry (DSC) analysis

Differential scanning calorimetry (DSC-Q600, IndiaMart, India) was performed for ART, the physical mixture of the lipids, and the prepared niosomes. In this regard, 50 mg of each sample was utilized. The scan rate was set at 10 °C/min, and the samples were heated from 25 °C up to 100, 90, and 200 °C for ART, niosomes, and lipid mixture, respectively.

#### Drug release study

Drug release was performed through the centrifugation ultrafiltration technique^58^. In this regard, 750 μL of ART-loaded niosomes with an average diameter of 100 nm was preliminary mixed with 4250 µL of the release medium (which contained 80%v/v phosphate buffer saline (PBS), pH 7.4 and 20%v/v ethanol) to maintain the sink condition. The mixture was placed in a shaker-incubator at 100 rpm at 37 °C, and the samples were taken after 1, 3, 7, and 24 h. The prepared samples were poured into the Amicon filter tubes and centrifuged at 4000 rpm for 5 min, and the filtrate was analyzed. The amount of the released drug was calculated, and the results were compared with the permeation of the free drug. Moreover, the drug release kinetics and the most probable mechanism of drug release from the niosomes were assessed.

### In vitro anti-leishmanial effects against the promastigotes of *L. major*

*L. major* standard strain (Mcan/IR/07/Moheb/-gh) was obtained from the infected Balb/c mice from the Department of Parasitology and Mycology, Shiraz University of Medical Sciences, Shiraz, Iran. Since the promastigote is an extracellular form of the parasite, therefore the specific cytotoxicity of the designed formulation was assessed on promastigotes. The promastigotes were cultured in RPMI 1640 medium enriched with FBS (15%v/v), penicillin (100 IU/ml), and streptomycin (100 µg/ml) and then incubated at 24–26 °C. The stationary phase promastigotes were sub-cultured to produce a higher number of parasites^59^. The obtained parasites were specifically used for each experiment.

Anti-leishmanial effects of ART solution (prepared from the standard powder of ART), drug-free niosomes, and ART-loaded niosomes with average diameters of 100 and 300 nm were assessed through the MTT assay test^60^, and the results were compared with L-AMB (AmBisome®, Gilead Sciences Co., California, USA) as the positive control and untreated well as the negative control. ART-loaded niosomes with average diameters of 100 and 300 nm were selected to assess the effect of particle size on cell internalization. For this test, 100 µL of parasite suspension at a concentration of 1 × 10^6^ parasite/mL was added to a 96-well microplate; then, 100 µL of ART, drug-free niosomes, ART-loaded niosomes, and L-AMB at concentrations of 2.5, 5, 10, 20, 50, 100, 250, 500, and 1000 µg/mL were poured into the wells, and the microplate was incubated at 25 °C for 24 and 48 h. Then, cell viability was assessed by the addition of 20 µL of MTT (5 mg/mL), and the samples were kept in a dark place for 24 h. After that, DMSO was added, and the absorbance of the samples was measured after 20 min using an ELISA plate reader (Biotek, Spain) at 570 nm^20^. All experiments were done in triplicate.

### Cytotoxicity assessment using J774 cell line

General cytotoxicity of different formulations was assessed using intact (uninfected) macrophage cells (J774 cell lines). In this regard, 2 × 10^4^ cells were seeded in a 96-well microplate and incubated at 37 °C for 24 h^20^. To remove the unattached cells, the supernatant was removed and replaced with fresh RPMI 1640 medium which contained 100 µL of 2.5, 5, 10, 20, 50, 100, 250, 500, and 1000 µg/mL of ART solution, drug-free niosomes, ART-niosomes, and L-AMB. Then, the samples were incubated at 37 °C and 5% CO_2_ for 24 and 48 h. After that, the cell viability and cell cytotoxicity were determined through the MTT colorimetric test^61^. Cell viability and cytotoxicity were calculated using Eqs. (4) and (5), respectively. In this study, untreated cells and L-AMB were considered as negative and positive controls, respectively.

4 $$\%Cell viability=\frac{OD test-OD blank}{OD control-OD blank}\times 100$$

5 $$\%Cytotoxicity=100-\%Cell viability$$

### Animal study

The animal study was designed to evaluate the anti-parasite effect of the designed formulation in amastigotes. BALB/c mice were obtained from the Center of Experimental and Comparative Medicine Center at Shiraz University of Medical Sciences, Shiraz, Iran. The BALB/c mice, with an average weight of 20–25 g, were kept in a relative humidity of 40% and temperature of 25 °C. Animals were handled according to the protocol of the Ethics Committee of Shiraz University of Medical Sciences, Shiraz, Iran, with Ethics Code No. IR.SUMS.REC.1401.307, and approval date of 07.31.2022. The reporting in the manuscript follows the recommendations in the ARRIVE guidelines.

#### In vivo anti-leishmanial effects using animal model

BALB/c mice with leishmanial wounds were randomly divided into 5 groups, each consisting of 5 mice. These 5 groups were treated with ART conventional gel 1%, ART niosomal gel 1%, drug-free niosomal gel, drug-free conventional gel, and topical nanoliposomal amphotericin B gel 0.4% (SinaAmpholeish®). Carbomer 934 polymer at a concentration of 0.5%w/w was used in the preparation of ART conventional gel, ART niosomal gel, drug-free niosomal gel, and drug-free conventional gel. The anti-leishmanial effects of the prepared formulations were compared with the topical nanoliposomal amphotericin B gel (SinaAmpholeish®), as the positive control, which is commercially available in the Iranian pharmaceutical market and is approved for CL management. The aforementioned formulations were applied to the various treatment groups of mice, once daily for 28 days. Response to therapy was assessed through the measurement of the diameter of the wounds weekly for up to 4 weeks. The size of wounds in each group was assessed prior to treatment and then weekly for up to 4 weeks. The images of the leishmanial lesions were captured, and their sizes were estimated using Digimizer software (Version 5.4.9).

### Ethics declarations

Animals were handled according to the protocol of the Ethics Committee of Shiraz University of Medical Sciences, Shiraz, Iran, with Ethics Code No. IR.SUMS.REC.1401.307, and approval date of 07.31.2022. The reporting in the manuscript follows the recommendations in the ARRIVE guidelines.

### Supplementary Information

Supplementary Information.

## Data Availability

The data that support the findings of this study are available from [Soliman Mohammadi-Samani] upon request.
