# Supplementary material for: Preparation and characterization of artemether-loaded niosomes in Leishmania major-induced cutaneous leishmaniasis
Source: Sci Rep. 2024 May 2;14:10073. doi: 10.1038/s41598-024-60883-0 (PMC11065877; doi:10.1038/s41598-024-60883-0)
Supplement: Supplementary file 1 — Supplementary Information. [file 41598_2024_60883_MOESM1_ESM.pdf]

Preparation and Characterization of Artemether-Loaded Niosomes in *Leishmania major*-Induced Cutaneous Leishmaniasis

Uranous Niroumand[1,2], Mohammad Hossein Motazedian[3], Fatemeh Ahmadi[4], Qasem Asgari[3], Mohammad Saleh Bahreini[3], Parisa Ghasemiyeh[5], and Soliman Mohammadi-Samani[1,4,5]*

1. Department of Pharmaceutical Nanotechnology, School of Pharmacy, Shiraz University of Medical Sciences, Shiraz, Iran.
2. Student Research Committee, Shiraz University of Medical Sciences, Shiraz, Iran.
3. Department of Parasitology, School of Medicine, Shiraz University of Medical Sciences, Shiraz, Iran.
4. Department of Pharmaceutics, School of Pharmacy, Shiraz University of Medical Sciences, Shiraz, Iran.
5. Pharmaceutical Sciences Research Center, Shiraz University of Medical Sciences, Shiraz, Iran.

***Corresponding author: Soliman Mohammadi-Samani** (smsamani@sums.ac.ir), Pharm.D., Ph.D., Professor of Pharmaceutics, School of Pharmacy, Shiraz University of Medical Sciences, Shiraz, Iran.

Supplementary Table 1. The preliminary study to achieve the optimum ratio of lipid matrix including Triolein, Capryol PGMC, and cholesterol

	**Lipid matrix**			**Particle size (nm)**
	**Triolein**	**Capryol PGMC**	**Cholesterol**	
F1	0	0	100%	185
F2	0	100%	0	756
F3	100%	0	0	538
F4	50%	50%	0%	300
F5	50%	0	50%	217
**F6**	**25%**	**25%**	**50%**	**145**

Supplementary Table 2. Artemether release kinetics from niosomes with an average diameter of 100 nm

**Model**	**R-squared**
First-order	0.950
Zero-order	0.824
Higuchi	0.932
Korsmeyer-Peppas	0.986<br>(n = 0.169)

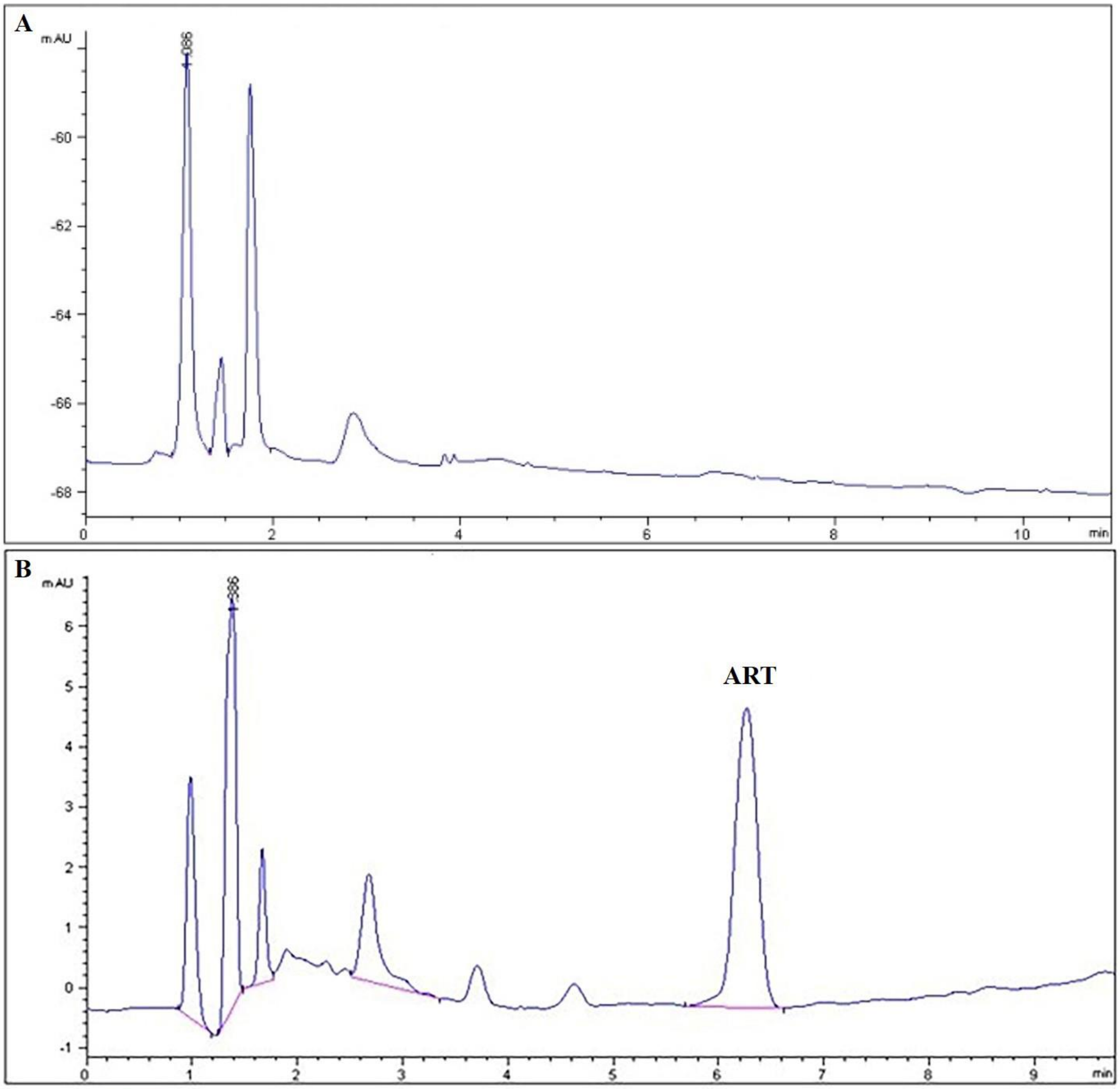

Supplementary Figure 1. A: A representative chromatogram of blank sample without drug, B: Chromatogram of artemether (ART) analyzed through the RP-HPLC method (ART concentration of 100 µg/ml; retention time of 6.2 min)

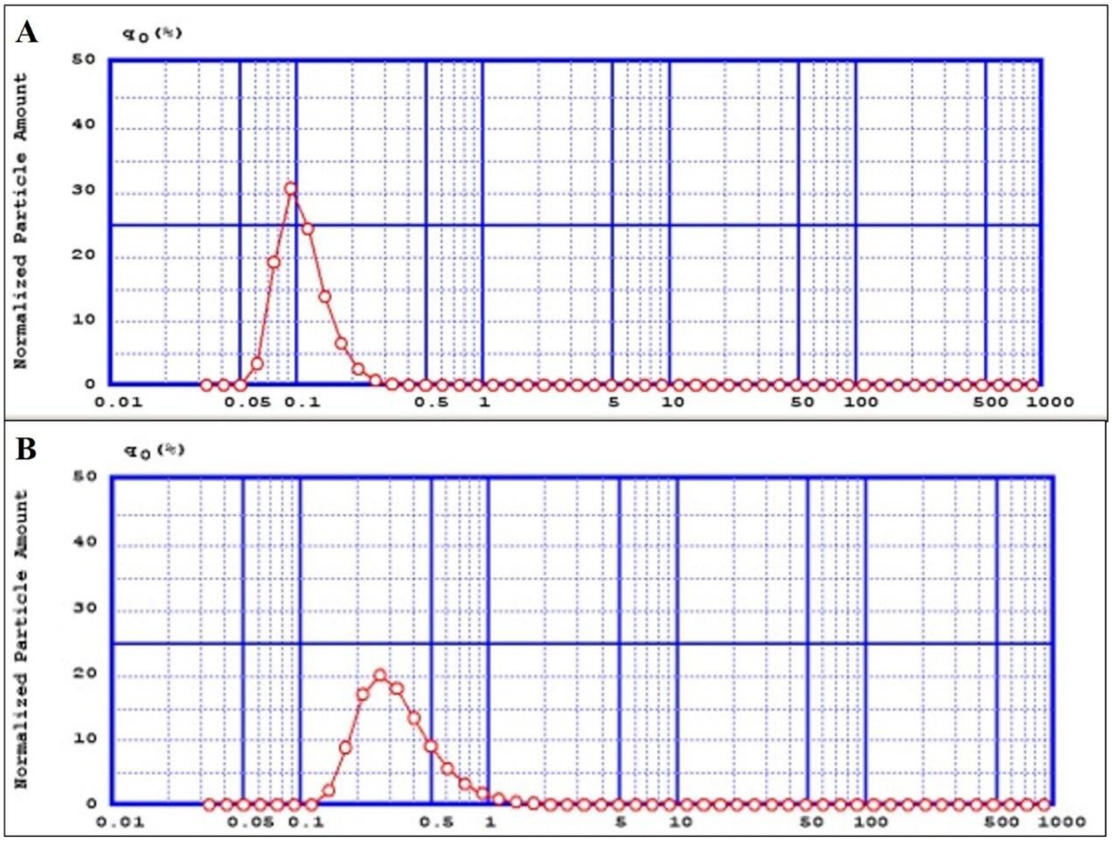

Supplementary Figure 2. A: Particle size analyzer (PSA) graph of artemether (ART)-loaded niosomes with an average particle size of 103±2 nm and span index of 0.9, B: PSA graph of ART-loaded niosomes with an average particle size of 314±1.5 nm and span index of 0.266.

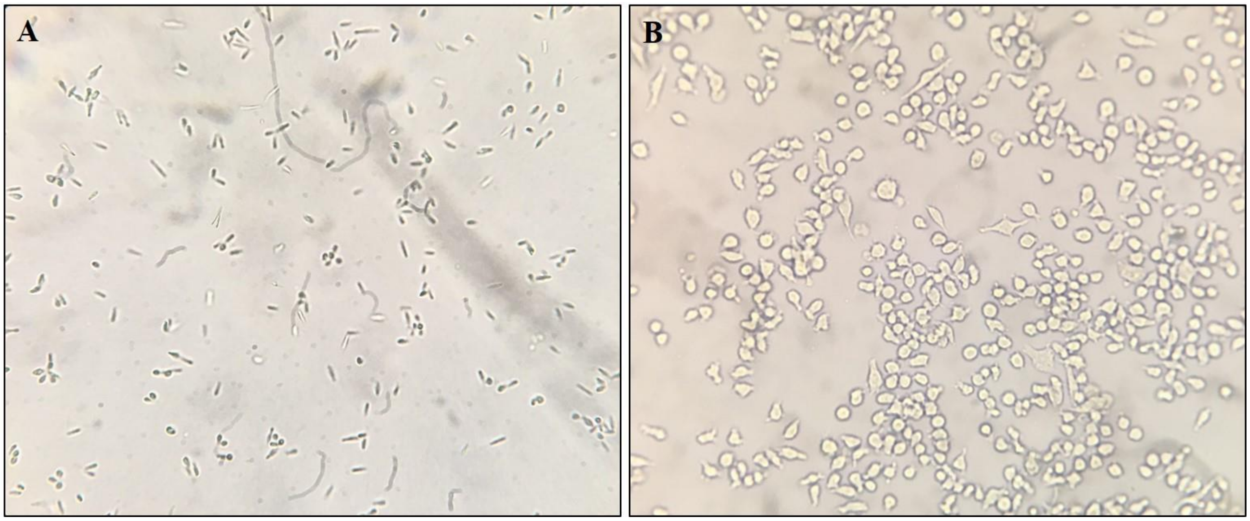

Supplementary Figure 3. A: Leishmania promastigotes used for *in vitro* cytotoxicity assessment and B: J774 macrophage cells used for general toxicity assessment

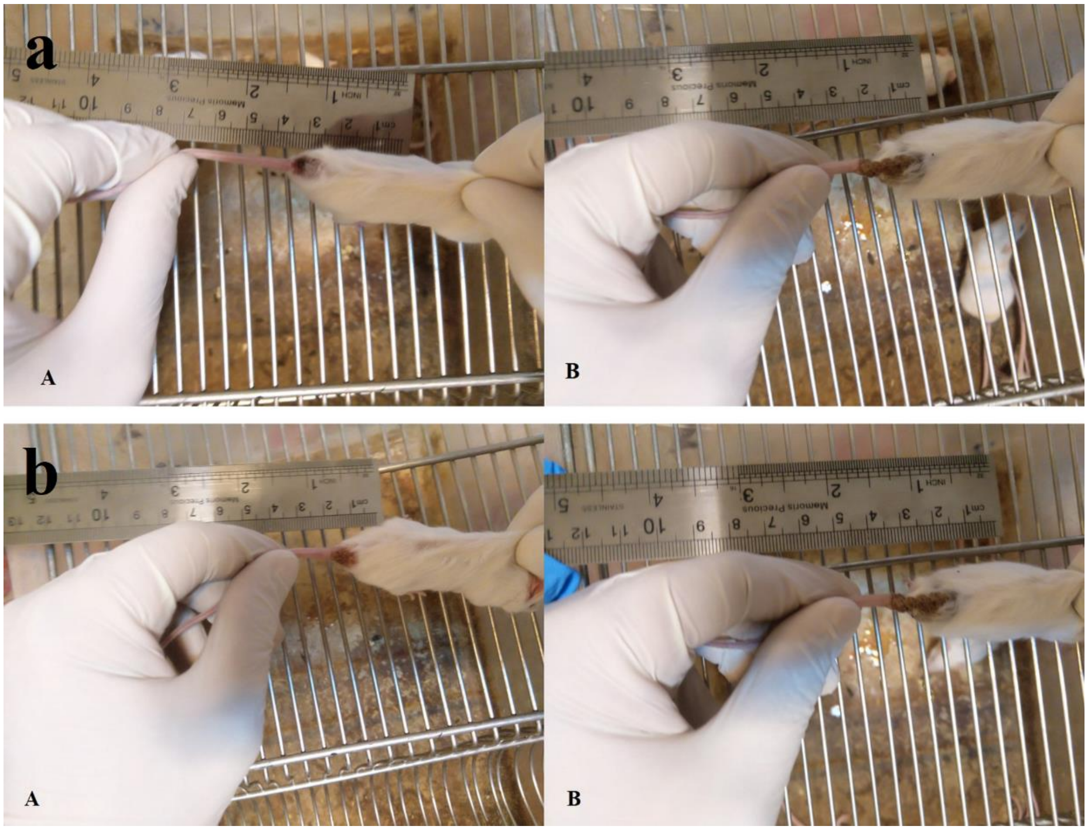

Supplementary Figure 4. Photographs of leishmanial wounds before (A) and after (B) topical treatment course (once daily for 28 days) with a: Drug-free conventional gel and b: Drug-free niosomal gel as negative controls

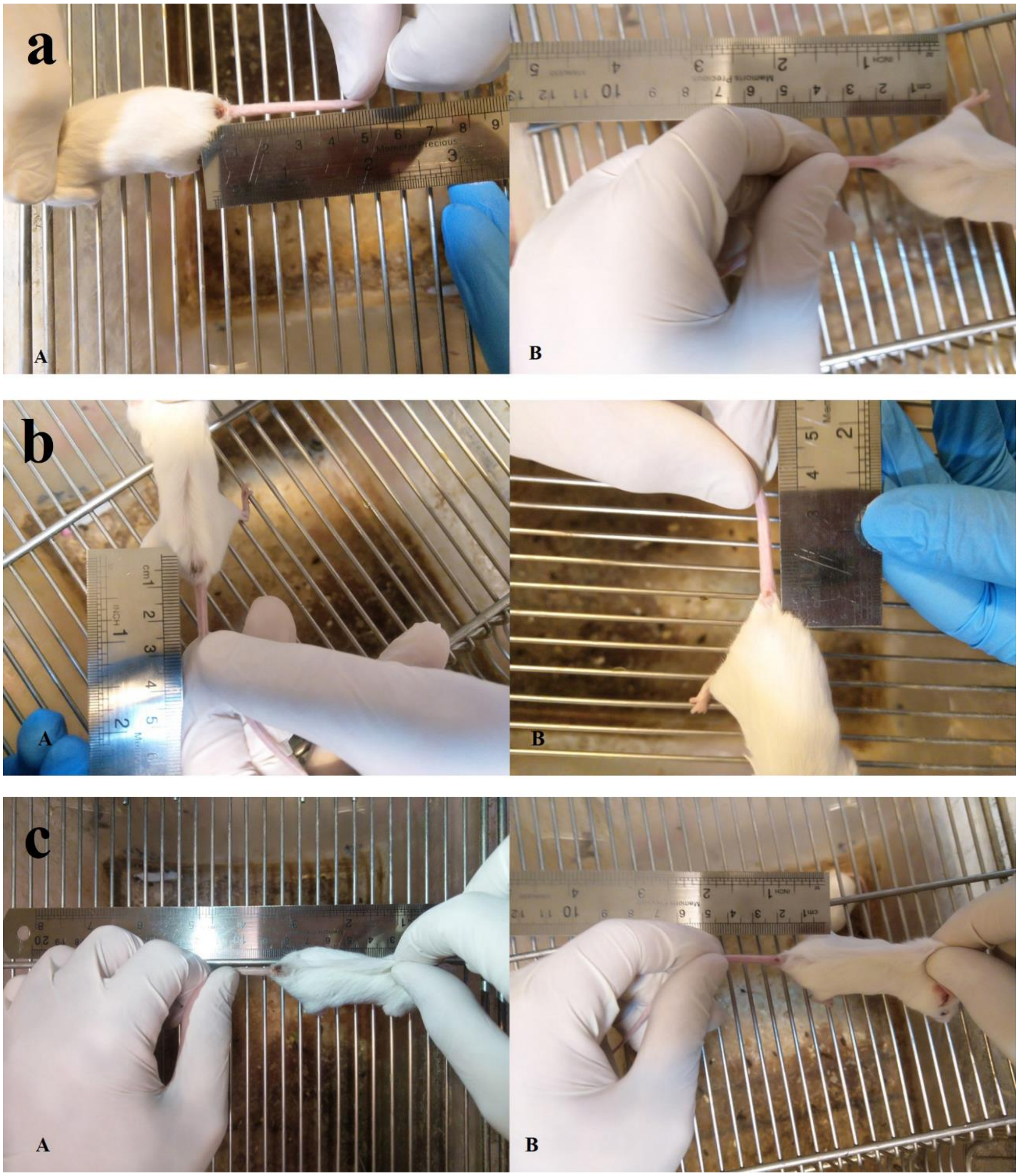

Supplementary Figure 5. Photographs of leishmanial wounds before (A) and after (B) topical treatment course (once daily for 28 days) with a: Niosomal artemether (ART) gel 1%, b: Liposomal amphotericin B gel 0.4% (SinaAmpholeish®), and c: Conventionlal ART gel 1%
